# Influence of therapeutic ultrasound on the biomechanical characteristics of the skin

**DOI:** 10.1186/s40349-016-0065-8

**Published:** 2016-08-17

**Authors:** Lígia Brancalion Catapani, Adriana da Costa Gonçalves, Nathalia Morano Candeloro, Lídia Aparecida Rossi, Elaine Caldeira de Oliveira Guirro

**Affiliations:** 1 Department of Biomechanics, Medicine and Rehabilitation of the Locomotor System, Ribeirão Preto Medical School, University of São Paulo, Ribeirão Preto, São Paulo Brazil; 2 Postgraduation Program in Rehabilitation and Functional Performance, University of São Paulo, 3900 Bandeirantes Avenue, 14049-900 Ribeirão Preto, São Paulo Brazil; 3 Ribeirão Preto College of Nursing, University of São Paulo, Ribeirão Preto, Brazil

**Keywords:** Skin, Elasticity, Therapeutic ultrasound

## Abstract

**Background:**

Skin function is dependent on its biomechanical characteristics, resistance, malleability, and elasticity. Therapeutic ultrasound may increase cutaneous malleability thus and optimize the rehabilitation process on specific diseases. The aim of this study is to evaluate possible alterations of biomechanical characteristics of the normal skin after therapeutic ultrasound application.

**Methods:**

Thirty-one volunteers took part of the study, and the average age was 31.61 ± 8.37 years old. Biomechanical characteristics evaluation of the skin was performed with the Cutometer *MPA* 580 (Courage + Khazaka Electronic—Köln, Germany) of 2-mm probe hole and 500-mbar vacuum. Skin characteristics were analyzed before and after therapeutic ultrasound application, and the variables R0 (distensibility), R2 (gross elasticity), and R6 (viscoelasticity) were used for the study. Areas of therapeutic ultrasound application (continuous, 3 MHz, 1 W/cm^2^ SATA) were defined at the upper limbs and standardized using a neoprene template. Sociodemographic data of volunteers were analyzed using SPSS 15.0. To analyze the distribution of the data, the Shapiro-Wilk test was used, which showed the normal distribution for R0 values, R2 and R6. For this procedure, the PROC TTEST from SAS® 9.0 software and Minitab 16 software, with significance, was set at the 0.05 level.

**Results:**

In relation to R0, a significant increase (*p* = 0.001) was observed for the distensibility, when compared to values of pre- (0.3273 mm) and immediately post- (0.3795 mm) resource application which feature a greater distensibility. Related to R2 values, a significant increase (*p* = .001) of the gross elasticity at pre- (0.8419) and post- (0.8884) therapeutic ultrasound application was found.

**Conclusions:**

Therapeutic ultrasound promotes significant alterations of the biomechanical characteristics of the skin.

**Trial registration:**

ClinicalTrials.gov, 1111-1146-7342

## Background

The skin, the largest organ in the human body, performs multiple functions [1], including mechanical effects on tissue elasticity [2], a characteristic that is responsible for the return of the skin to its original form after tension removal [3]. It may vary according to the age, evaluated tissue composition, among others [4].

Biomechanical characteristics of tissues have been widely used as a characterization of these important since many pathological and physiological changes involving tissue. Properties such as elasticity, viscoelasticity, and distensibility which can be evaluated objectively are essential for the detection of skin disorders [5–7].

Ultrasonic energy is frequently used in soft tissue treatment, which causes therapeutic responses related to thermal and non-thermal (mechanical) effects [8–11].

The therapeutic ultrasound induces physiological changes in the skin [12], and this may lead to alterations of the extensibility of tissues through modifications in collagen properties by the thermal effects of therapeutic ultrasound [13–15].

Thermal ultrasound effect can be effective in increasing extensibility of collagen, thus aiding joint mobilization and stretching [11, 16].

The therapeutic ultrasound physical parameters that are best able to increase tissue malleability, especially skin malleability, remain to be established. There is a discrepancy related to the effects of therapeutic ultrasound at the collagen tissue extensibility between in vivo and in vitro studies [17], after the therapeutic ultrasound application. No studies were found that evaluate the influence of ultrasound on biomechanical characteristics of normal skin. The present study aims to elucidate the biological effects induced by therapeutic ultrasound on the skin and support the therapeutic intervention in tissue dysfunction.

## Methods

### Sample

Thirty-one volunteers took part of the study; the average age was 31.61 (±8.37) years old, from both genders. Subjects who presented comorbidities or deformities that could affect the skin structure and function were excluded from the study.

### Instrumentation

Evaluation of biomechanical characteristics of the skin was performed by the Cutometer, model MPA 580 (Courage + Khazaka Electronic—Köln, Germany), a probe of 2-mm hole and 500 mbar of suction per second, before and after the therapeutic procedure. The Cutometer is a non-invasive tool that facilitates analysis and characterization of the functional state of the skin, as well as the measurement of responses initiated by different therapeutic resources [5, 6, 18–22].

**Fig. 1:**
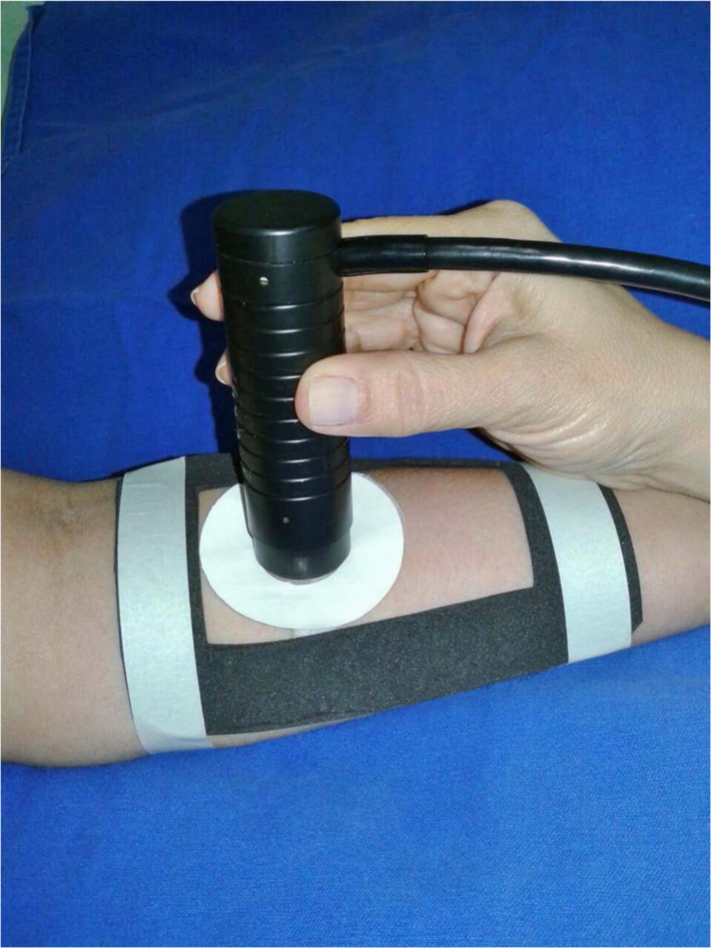
Cutometer probe application with specific adhesive rings fixed in the anterior forearm and delimited by a neoprene template

The evaluated zone was standardized and limited by a neoprene template of the 9 × 5cm area, cut at precisely two times the size of the effective radiating area of the ultrasound applicator onto the skin, and this served to restrict all treatments to the same size surface area [15, 23]. The areas established for evaluations with Cutometer bounded by specific adhesive rings for this purpose, positioned at the top edge of the mold to ensure the standardized location of the adhesives, removed for therapeutic interventions with the resources, and repositioned following (Fig. 1).

Measurements were performed after 20-min resting, controlled environment (temperature between 20 and 25 °C and humidity ranging 30–50 %), always in the morning in order to avoid interference in the chronobiological characteristics of the skin [24], and all assessments made in the anterior forearm because of the biomechanical characteristics of the skin vary in different regions [25]. The probe was maintained at the delimited area by 6 s, 500 mbar of suction, three times repetition, normalization of temperature interval, performed 15 min after skin contact [26], with representative displacement curves calculated by the Cutometer software [27–29].

**Fig. 2:**
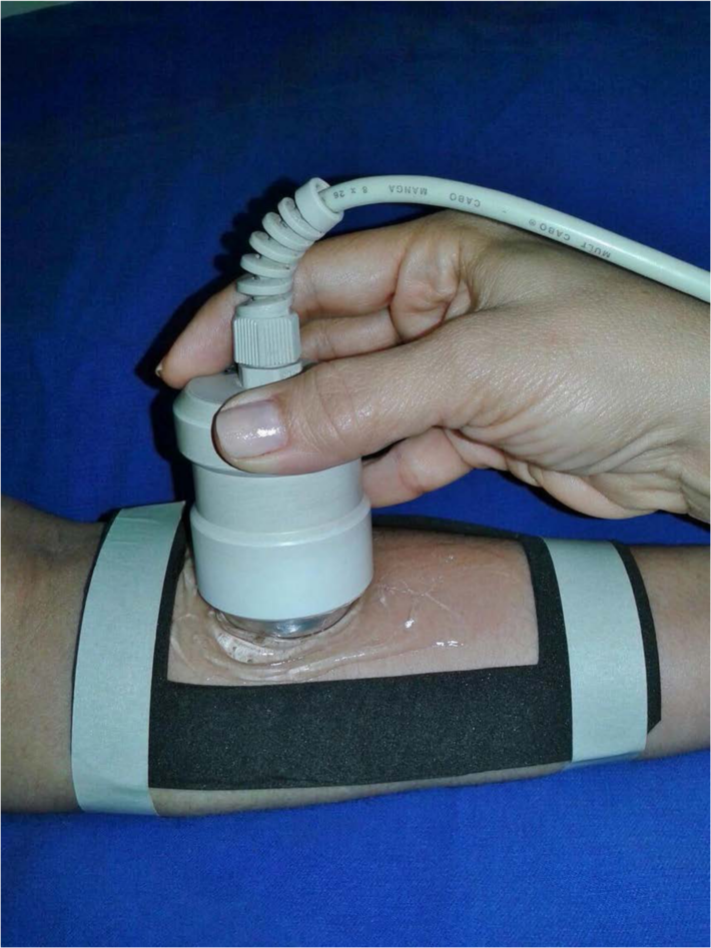
Application of therapeutic ultrasound in delimited area with neoprene template

The therapeutic intervention was performed with ultrasound Sonopulse III (IBRAMED-Amparo, Brazil) and it was periodically submitted to calibrations (irradiation pressure scale Ohmic UPMDT 10 model) [30]. The following parameters were employed: continuous mode, 3-MHz frequency, and 1 W/cm^2^ (SATA) intensity at the selected area and gel was used as a coupling agent. Irradiation time was 2 min per ERA (effective radiating area) of the transductor at both sides of the upper limbs, in a total of 4 min of application at a delimited area (Fig. 2).

Biomechanical characteristics analysis of the skin were performed in sequence and variables observed were the following: R0 - distensibility; R2 - gross elasticity (resistance against return capability of the tissue), which values close to one indicate greater elasticity, and R6 - viscoelasticity for which lower values indicate greater elasticity [31].

### Statistical analyses

Sociodemographic data of volunteers were analyzed using SPSS 15.0 (percentage, average with standard deviation, and median with minimum and maximum value).

To analyze the distribution of the data, the Shapiro-Wilk test was used, which showed the normal distribution for R0 values, R2 and R6. For this procedure, the PROC TTEST from SAS® 9.0 software was used in contribution to the Minitab 16 Software. Statistical significance was set at 0.05 level.

## Results

Sociodemographic features of the volunteers (*n* = 31) were the following: 31.61 (±8.37) years, the main gender was male (61.3 %, 19), youthful adult, 51.4 % (18) Caucasians, presenting the mean body mass index (BMI) of 25.44. From 31 upper limb areas evaluated, 22 (70.96 %) were arms, 6 (19.35 %) were forearms, and 3 (9.67 %) were the back of the hand.

Regarding biomechanical features of the skin pre- and post-therapeutic ultrasound (US) application, for R0 (distensibility, absolute value in mm), a significant increase of tissue distensibility could be noticed after therapeutic ultrasound application and the same was observed for R2 (gross elasticity, relative value) and R6 (viscoelasticity, relative value), being data respectively reported in Tables 1, 2, and 3.

**Table 1:** Values of the R0 variable before and after therapeutic ultrasound application at normal skin

Variable R0	Number	Average (mm)	Standard deviation (mm)	Standard error (mm)
Pre-intervention	31	0.3273	0.1038	0.0186
Post-intervention	31	0.3795*	0.1042	0.0187
Difference		−0.05226	0.05396	0.00969

*Differs from pre- (*p* = 0.001)

**Table 2:** Values of the R2 variable before and after therapeutic ultrasound application at normal skin

Variable R2	Number	Average	Standard deviation	Standard error
Pre-intervention	31	0.8419	0.0946	0.0170
Post-intervention	31	0.8884*	0.0624	0.0112
Difference		−0.0464	0.0675	0.0121

*Differs from pre- (*p* = 0.001)

**Table 3:** Values of the R6 variable before and after therapeutic ultrasound application at normal skin

Variable R6	Number	Average	Standard deviation	Standard error
Pre-intervention	31	0.4605	0.1079	0.0194
Post-intervention	31	0.3629*	0.0934	0.0168
Difference		0.0976	0.0827	0.0149

*Differs from pre- (*p* = 0.001)

**Fig. 3:**
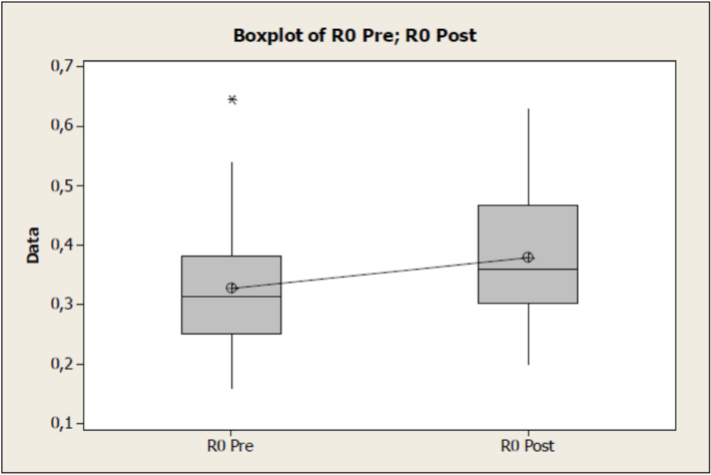
Values of the R0 (mm) variable before and after therapeutic ultrasound application on normal skin

Table 1 and Fig. 3 demonstrate 95 % significance level, with difference showed by values of R0 pre- and post-therapeutic ultrasound, considering *p* = 0.001 for the statistic test of *t* value = −5.39, with a reliability interval difference average (−0.07205; −0.03246).

**Fig. 4:**
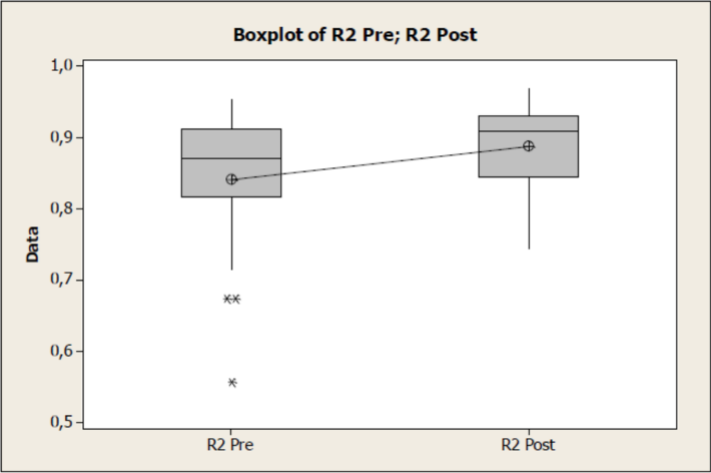
Values of the R2 variable before and after therapeutic ultrasound application on normal skin

Regarding to R2, relative value (Table 2) and Fig. 4, it is also possible to notice that for the same significance level of 95 %, there is evidence that R2 values before are different from those post-therapeutic ultrasound application, considering *p* = 0.001 for statistic test of *t* value = 3.83, with a reliability interval difference average (−0.0712; −0.0216).

**Fig. 5:**
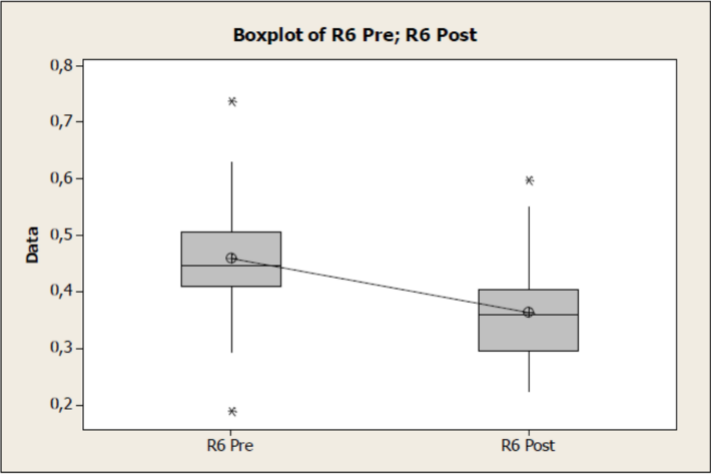
Values of the R6 variable before and after therapeutic ultrasound application on normal skin

The R6 variable indicated in Table 3 and Fig. 5, where it is considered *p* = 0.001 for a statistic *t* value test = 6.57 and a reliability interval of 95 % for difference in averages of 0.0672 and 0.1279, did not present significant deviations of normality.

## Discussion

No previous study has investigated the effect of therapeutic ultrasound (US) on biomechanical characteristics (distensibility, elasticity, and viscoelasticity) of the skin.

The effects inherent to the increase of malleability by therapeutic ultrasound are related to heating that is intensity-dependent, as well as to quantity of collagen fibers at the tissue submitted for treatment, whereupon the greater collagen quantity, the better is the absorption [23], with better efficiency at continuous mode [32].

Despite the ultrasound often be used in therapeutic procedures, inefficiencies highlighted in the results are probably due to wrong parameters, not considering the size of the area, duration, intensity, frequency, type of fabric, transducer movement, and the therapeutic window [16]. The present study utilized an application time of 4 min which was used in a bound area of approximately 10 cm^2^.

The absorption of ultrasound in tissues is determined by the protein constituents, being observed enhanced absorption in the tissue with more collagen [33], and skin dermis fibers are responsible for providing elasticity and also resistance to lengthening [34, 35].

Body segments set out for elasticity evaluation of the skin were the forearm, arm, and back of the hand which are considered easily accessible areas and relatively flat [36], optimizing the procedures performed. Measurements of biomechanical features of the skin were always done by the same observer, so it would be possible to standardize pressure at the probe; different pressures at the probe of Cutometer may produce different responses [31, 36–38].

The utilization of Cutometer equipment has been described previously, as an important and effective tool for objective and non-invasive measurements of biomechanical properties of the skin, yielding absolute and relative data. A study has used the Cutometer to evaluate the elasticity of the skin related to age, while the other has analyzed the skin elasticity in different parts of the body, and both studies found differences, which is the reason why it has been standardized to specific areas and age for evaluation [39, 40].

For the World Health Organization [41], a body mass index (BMI) lower than 18.5 indicates low weight; ranging between 18.5 and 24.9 indicates a normal weight and higher than 25 is considered overweight. In the present study, the average of the body mass index of volunteers fits in the overweight. However, there is controversy regarding correlations of skin elasticity and obesity rate.

The study [42] did not find a correlation between skin elasticity and obesity degree. However, Smalls [4] demonstrates a potential influence of body composition at biomechanical properties of the skin, the reason why those values were evaluated.

Other authors [43] have pointed that a single observer can safely utilize the Cutometer for measuring skin elasticity, as well as reported that R0 (Ue) may be used in an adequate way for measuring, assuming that it is one of the best variables to quantify skin elasticity, being R0 measurement related by authors [44] to plastic and elastic deformations.

Because lower values of R0 represent greater firmness, i.e., lower skin distensibility, it is possible to notice a significant increase of this tissue property after therapeutic ultrasound application at normal skin in the present study, even as observing the R2 variable, which has also demonstrated greater elasticity, proving the efficacy of therapeutic ultrasound reported on literature regarding to the increase of distensibility [42].

When skin elasticity resistance (Ue) is at the limit, sustained strength applied to the skin results in a greater deformation on it due to viscoelasticity (Uv) which moves the interstitial fluid through skin fibrous net [27]. The viscoelasticity represented by this variable showed a significant decrease of its initial average value. Considering that the lower the R6 value, the greater the elasticity, it is possible to notice an improvement of elasticity at normal skin after therapeutic ultrasound application, corroborating to the findings of authors who study the effects of therapeutic ultrasound regarding the increase of tissue elasticity [45].

The Cutometer can also be used to evaluate biomechanical properties of morbidities of the skin, such as burns and keloids. The anisotropic behavior of different tissues relative to the normal skin should be considered in the studies [46–48].

Feature-inherent effects of therapeutic ultrasound at the skin may guide studies that evaluate the effect of therapeutic resources at different morbidities.

The results of this study are limited to effects produced by a single application of therapeutic ultrasound. The assessment of the effects produced by chronic treatment can clarify the effects inherent to stimulation with the largest number of interventions with therapeutic ultrasound.

## Conclusions

Significant alterations on the biomechanical characteristics of the normal skin after therapeutic ultrasound application were found.

The effects of therapeutic resources on the different tissues are still a challenge to researchers; increased knowledge about the effects in different tissues is important in the treatment of different clinical conditions.

## Abbreviations

BMI: Body mass index
Mm: Millimeters
US: Ultrasound
